# Altered Red Blood Cell Fatty Acid and Serum Adipokine Profiles in Subjects with Obesity

**DOI:** 10.3390/biomedicines11123320

**Published:** 2023-12-15

**Authors:** Asier Léniz, Alfredo Fernández-Quintela, Sara Arranz, Kevin Portune, Itziar Tueros, Eunate Arana, Luis Castaño, Olaia Velasco, María P. Portillo

**Affiliations:** 1 Vitoria-Gasteiz Nursing School, Osakidetza-Basque Health Service, 01009 Vitoria-Gasteiz, Spain; asier.leniz@gmail.com; 2 BIOARABA Institute of Health, 01006 Vitoria-Gasteiz, Spain; mariapuy.portillo@ehu.eus; 3 CIBER Physiopathology of Obesity and Nutrition (CIBERobn), Institute of Health Carlos III, 01006 Vitoria-Gasteiz, Spain; 4 Nutrition and Obesity Group, Department of Nutrition and Food Sciences, Faculty of Pharmacy, University of the Basque Country (UPV/EHU), Paseo de la Universidad, 7, 01006 Vitoria-Gasteiz, Spain; 5 Lucio Lascaray Research Centre, Avenida Miguel de Unamuno, 3, 01006 Vitoria-Gasteiz, Spain; 6 Department Pharmacy and Food Sciences, Faculty of Pharmacy, University of the Basque Country (UPV/EHU), 01006 Vitoria-Gasteiz, Spain; 7 AZTI, Food Research, Basque Research and Technology Alliance (BRTA), Parque Tecnológico de Bizkaia, Astondo Bidea, Edificio 609, 48160 Derio, Spain; sarranz@azti.es (S.A.);; 8 Hospital Universitario Cruces, BIOBIZKAIA Institute of Health, 48903 Barakaldo, Spainolaia.velascovielba@bio-bizkaia.eus (O.V.); 9 Department Pediatrics, Faculty of Medicine and Nursing, University of the Basque Country (UPV/EHU), 48940 Leioa, Spain; 10 CIBER Diabetes and Associated Metabolic Diseases (CIBERdem), Institute of Health Carlos III, 48903 Barakaldo, Spain; 11 CIBER Rare Diseases (CIBERer), Institute of Health Carlos III, 48903 Barakaldo, Spain; 12 European Reference Network on Rare Endocrine Conditions (ENDO-ERN), 48903 Barakaldo, Spain

**Keywords:** obesity, adipokines, FGF21, NOV/CCN3, red blood cell fatty acid, regression analysis

## Abstract

Background: Adipokines, as well as the fatty acid profile of red blood cell (RBC) membranes, are known to play important roles in the development and progression of metabolic complications induced by obesity. Thus, the objective of this study is to compare the serum adipokine profile and the RBC membrane fatty acid profile of normal-weight and obese adults, and to analyze their relationship with serum biochemical parameters. Methods: An observational case–control study was performed in 75 normal-weight and obese adult subjects. Biochemical serum parameters, eight serum adipokines and the RBC membrane fatty acid profiles were measured. Associations between parameters were established using regression analysis. Results: Subjects with obesity showed increased levels of leptin, fibroblast growth factor 21 (FGF21) and overexpressed nephroblastoma (NOV/CCN3), decreased adiponectin, and similar levels of vaspin and chemerin compared to normal-weight subjects. Significant positive and negative correlations were found with triglycerides and high-density lipoprotein-cholesterol (HDL-c), respectively. An increase in the total ω-6 fatty acids in the RBC membrane fatty acid profiles in subjects with obesity was observed, because of higher levels of both dihomo-γ-linolenic acid (DGLA) and arachidonic acid (AA), and decreased total ω-3 fatty acids, mainly due to lower levels of docosahexaenoic acid (DHA). The ω-6/ω-3 ratio in the RBCs was significantly higher, suggesting an inflammatory status, as was also suggested by a reduced adiponectin level. A negative association between DGLA and adiponectin, and a positive association between DHA and serum triglycerides, was observed. Conclusions: Important alterations in serum adipokine and RBC fatty acid profiles are found in subjects with obesity.

## 1. Introduction

Obesity can be defined as an excessive accumulation of fat mass with consequential effects on health. Nowadays, obesity has become a relevant health problem worldwide, especially in Western countries, and it affects almost 30% of the world population [1]. This disease is a key factor in the development of metabolic syndromes, and it can induce alterations at the inflammatory and hormonal level. As a result, obesity is associated with a variety of co-morbidities such as type 2 diabetes, dyslipidemia, hypertension, arthritis, non-alcoholic fatty liver diseases, breathing pathologies and some types of cancer [2]. 

Cytokines are 5 to 30 kDa peptides, glycoproteins or proteins secreted by cells that act, via receptors, to regulate the growth, responsiveness or maturation of certain target cell groups. Once a cytokine binds to its corresponding receptor in the cell surface, an intracellular signaling cascade is triggered, which leads to changes in gene expression of the target cell and, consequently, to a biological action. Cytokines can regulate complex networks with autocrine, paracrine or endocrine functions and can act in a pleiotropic, redundant, synergic or antagonistic manner [3].

Among cytokines, great attention has been paid to those secreted by white adipose tissue, known as adipocytokines or adipokines. Adipose tissue is considered an endocrine organ that can establish a crosstalk with other organs and tissues by secreting adipokines. These molecules play important roles in regulating whole-body homeostasis with a relevant influence in the development and progression of metabolic complications induced by obesity [4,5]. In the presence of adipocyte hypertrophy produced by excess energy storage, adipokine secretion is altered due to an energy imbalance, which contributes to the pathogenesis of obesity-associated complications [6].

On the other hand, the fatty acid profile of red blood cell (RBC) membranes can be considered a good predictor of the fatty acid profile of other body tissues [7], and can help monitor the ω-6 and ω-3 fatty acid contents in phospholipids that are directly linked with inflammation mediators. Additionally, since RBC half-life is around 4 months, membrane fatty acid composition of these cells can reflect the status of obesity-related diseases [8].

The aim of the present study is to compare the serum adipokine profile and the RBC membrane fatty acid profiles of normal-weight and obese adults, and to analyze their relationship with serum biochemical parameters. 

## 2. Materials and Methods

### 2.1. Design and Subjects

The present observational case–control study was carried out on a cohort of 37 normal-weight (18 males and 19 females) and 38 obese (19 males and 19 females) 19–68 year-old adult subjects recruited from the Endocrinology Department at the Hospital Universitario Cruces (Barakaldo, Spain). The distribution between both groups was made according to body mass index (BMI), taking BMI > 30 kg/m^2^ as a reference to classify obesity and 18.5 < BMI < 25 kg/m^2^ for the normal-weight group. After a physical examination by an endocrinologist, participants with any kind of acute or chronic disease or who were taking medication were excluded from the study. Anthropometric measurements were all conducted by doctors during the participant’s visit to the Hospital Universitario Cruces/IIS Biocruces Bizkaia.

The study protocol was approved by the Euskadi Clinical Research Ethics Committee (permission number PI2016181) and carried out according to the ethics principles derived from the Declaration of Helsinki, consistent with Good Clinical Practice guidelines. All participants were provided with a written informed consent form, in agreement with the corresponding laws (Organic Law 3/2018, of December 5, Protection of Personal Data and guarantee of digital rights; Law 14/2007 on Biomedical Research and RD 1716/2011 of Biobanks).

### 2.2. Blood Collection and Biochemical Analysis

Blood samples were obtained in vacutainer tubes containing ethylenediaminetetraacetic acid (EDTA). Blood was centrifuged at 1250× *g* for 20 min, and the plasma obtained was stored at −80 °C until analysis. Plasma concentrations of glucose, insulin, total cholesterol (TC), high-density lipoprotein cholesterol (HDL-c), low-density lipoprotein cholesterol (LDL-c), triglycerides (TG), aspartate aminotransferase (AST), alanine aminotransferase (ALT), uric acid and bilirubin were measured using standard laboratory assays.

For cytokine measurements, sensitive and specific enzyme-linked immunoassay kits were used: RD191001100 (BioVendor, Brno, Czech Republic) for leptin, RD195023100 (BioVendor, Brno, Czech Republic) for adiponectin, RD191100200R (BioVendor, Brno, Czech Republic) for omentin, ab155430 (Abcam, Cambridge, UK) for chemerin, RD191097200R (BioVendor, Brno, Czech Republic) for vaspin, ab193710 (Abcam, Cambridge, UK) for NOV-CCN3, ab222506 (Abcam, Cambridge, UK) for FGF21 and RD191016100 (BioVendor, Brno, Czech Republic) for resistin. The measurements were performed with a Labsystems iEMS Reader MF analyzer plate photometer (Labsystems Diagnostics Oy, Helsinki, Finland).

The homeostatic model assessment for insulin resistance (HOMA-IR) was calculated using the following formula:

HOMA-IR = Fasting plasma insulin (μU/mL) × fasting glycaemia (mg/d μU L)/405

### 2.3. Red Blood Cell (RBC) Membrane Fatty Acid Analysis 

The fatty acid composition of mature RBC membrane phospholipids was obtained from blood samples (approximately 2 mL) collected in vacutainer tubes containing ethylenediaminetetraacetic acid (EDTA). Samples were shipped to the Lipidomic Laboratory, where they underwent the certified procedure MEM_LIP_1 upon arrival, according to the quality control guidelines. The absence of hemolysis was promptly checked. For the blood, the procedure to extract and work-up lipids to obtain fatty acid methyl esters (FAMEs) was conducted following an automated protocol, including the selection of mature RBCs as previously disclosed [8,9]. Briefly, the EDTA blood was centrifuged (4000 rpm for 5 min at 4 °C) and the cell fraction was isolated based on the high density of the aged cells and controlled using a cell counter (Scepter 2.0 with Scepter™ Software Pro, EMD Millipore, Darmstadt, Germany). The automated equipment subsequently executed the following tasks: cell lysis, membrane pellets isolation, and phospholipid extraction from pellets using the Bligh and Dyer method [10], transesterification to FAMEs was made with a solution of methyl alcohol and potassium hydroxide (0.5 mol/L) during 10 min at room temperature and extracted using n-hexane (2 mL). The automated procedure is available at the Lipidomic Laboratory facility of Lipinutragen srl (www.lipinutragen.it) and is compliant with the ISO/EIC 17025:2017 regulation [11]. Laboratory machinery used to analyze FAMEs were a capillary column gas chromatography Agilent 6850 Network, supplied with a fused silica capillary column Agilent DB23 (60 m × 0.25 mm × 0.25 µm) and a flame ionization detector (FID). Libraries of *trans* isomers of monounsaturated (MUFAs) and polyunsaturated fatty acids (PUFAs) [12] and commercial standards allow us to separate and identify fatty acids as well as their geometrical and positional isomers registered. The results are reported as a relative % for each fatty acid, with more than 97% of the GC peaks recognized with appropriate standards.

### 2.4. Statistical Analysis 

Data are presented as mean values ± standard error of the mean. The statistical analyses were performed using IBM SPSS statistics (v25, Chicago, IL, USA). Normal data distribution were assessed with Shapiro–Wilk’s test and the Kolmogorov–Smirnov test. Differences between groups (subjects with a normal weight and subjects with obesity) were evaluated using Student’s *t* test or the Mann–Whitney U test for data that were not normally distributed. Correlations between variables were conducted using Spearman’s rank correlation. Multiple linear regression analysis was used to find the major determinants for serum biochemical parameters. This stepwise regression was achieved by including all potential independent variables in the model and eliminating those that were not statistically significant using a backward selection method. Statistical significance was chosen at *p* value of <0.05.

## 3. Results

### 3.1. Anthropometric Parameters

As expected, obese subjects presented higher values of BMI and waist circumference (Table 1). 

### 3.2. Biochemical Parameters 

Table 2 summarizes the biochemical parameters of normal-weight subjects and subjects with obesity. The latter presented higher concentrations of glucose, insulin, uric acid, LDL-cholesterol, triglycerides and alanine aminotransferase (ALT/GPT) than normal-weight subjects, as well as lower levels of HDL-cholesterol and albumin. No significant differences were found in total cholesterol and aspartate aminotransferase (AST/GOT). The HOMA-IR index was significantly higher in subjects with obesity.

### 3.3. Adipokine Concentrations

**Figure 1:**
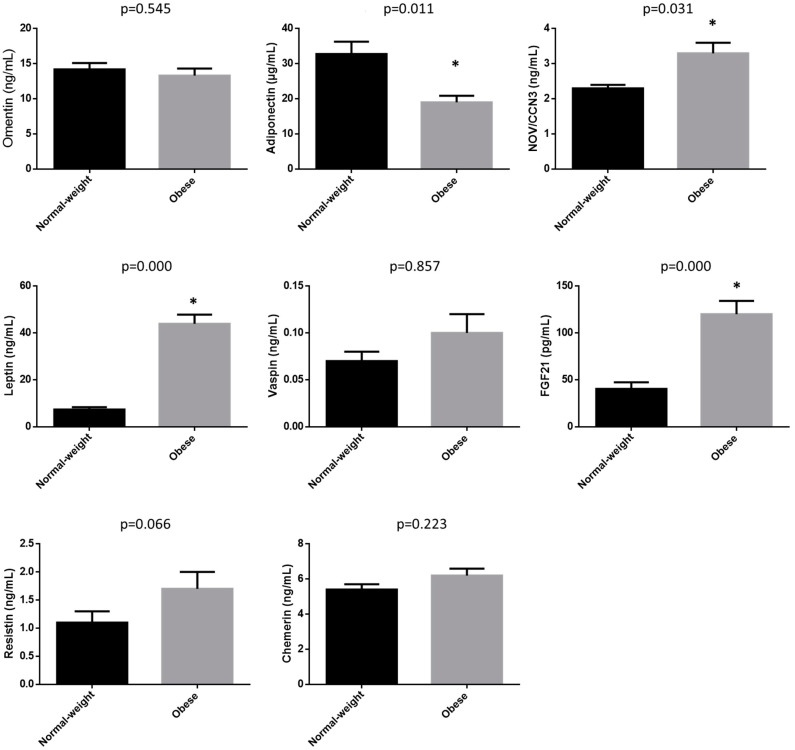
Adipokine concentrations in normal-weight subjects and subjects with obesity. Data are expressed as mean ± SEM. * Statistically different from the normal-weight group.

Figure 1 shows adipokine concentrations. Subjects with obesity showed significantly higher values of NOV/CCN3, leptin and FGF21, and significantly lower values of adiponectin than normal-weight subjects. No significant differences were found in omentin, vaspin and chemerin. Lastly, resistin showed a tendency towards higher values in subjects with obesity than in individuals with normal weight. 

### 3.4. Fatty Acid Profile of RBCs

Table 3 shows the fatty acid profile of RBCs. No significant differences were observed in saturated fatty acids between both groups. Although individual monounsaturated fatty acids (MUFAs) were not different between groups, subjects with obesity showed significantly lower levels of total MUFAs. The fatty acid that contributed the most to this difference was sapienic acid (C16:1, 6c). The ratio of saturated/monounsaturated fatty acid (SFAs/MUFAs) was significantly higher in obese subjects compared to normal-weight subjects, mainly due to the lower level of MUFAs. Subjects with obesity also showed a significantly lower level of total ω-3 fatty acids, caused for the most part by the lower level of docosahexaenoic acid (DHA), and an increased level of total ω-6 fatty acids which was largely induced by the increase in dihomo-γ-linolenic acid (DGLA) and arachidonic acid (AA). Consequently, the ratio ω-6/ω-3 was significantly higher in individuals with obesity than in normal-weight subjects. 

Based on the ratios between specific fatty acids, the activity of delta-5 desaturase (∆5D), delta-9 desaturase (∆9D) and delta-6 desaturase (∆6D) + elongase (ELO) was estimated. Whereas a nonsignificant difference was observed in the activity of ∆9D, that of ∆5D was higher in subjects with obesity and ∆6D + ELO was lower in subjects with obesity.

### 3.5. Correlation Analysis

The correlations between serum metabolic parameters and cytokines, using only cytokine data that showed significant differences between non-obese subjects and individuals with obesity, are shown in Table 4. Positive correlations are as follows: Serum glucose, insulin and HOMA-IR with leptin and FGF21; Serum triglycerides with leptin; HDL-c with adiponectin and FGF21; Uric acid with FGF21; ALT/GPT with FGF21. In contrast, Serum glucose, insulin and HOMA-IR, serum triglycerides, uric acid, and ALT/GPT all correlated negatively with adiponectin.

Lastly, Table 5 shows the correlations between cytokines and RBCs fatty acid profiles. Adiponectin was positively correlated with ∆6D + ELO and ∆5D, and negatively correlated with DGLA. Negative correlations were observed between leptin, ∆6D + ELO and DHA and total ω-3 fatty acids, and a positive correlation between this adipokine and the ratio ω-6/ω-3. FGF21 was positively correlated with DGLA and negatively with ∆6D + ELO. 

### 3.6. Stepwise Multiple Regression Analysis for Major Determinations

This analysis was performed to find a set of independent variables that significantly influence biochemical parameters, adipokines and RBC membrane fatty acid. The independent variables included in the multiple regression analysis were participant sex, age and BMI, for all dependent variables, the parameters that showed to be correlated with each dependent factor through backward stepwise selection. The results are shown in Table 6. 

## 4. Discussion

Cytokines can represent interesting biomarkers of metabolic disorders and diseases. In the present study the cytokine profile of subjects with obesity that showed metabolic syndrome, in agreement with the US National Cholesterol Education Programme Adult Treatment Panel III (NCEP ATP III) [13], was evaluated and compared with that of healthy normal-weight subjects. Subjects with obesity showed an altered serum cytokine profile, characterized by increased levels of leptin, FGF21 and NOV/CCN3, a decreased level of adiponectin, and similar levels of vaspin and chemerin compared to normal-weight subjects. In general terms, this altered profile can be related to an increased risk of suffering alterations in glycemic control and inflammation [5,14]. The alterations in adipokine levels in subjects with obesity have been widely studied. However, in the vast majority of studies either a single adipokine or a reduced number of adipokines has been analyzed. Thus, a strength of the present research is that concurrent levels of eight adipokines have been studied in the same cohort together with the association with other metabolic parameters such as RBC fatty acids and biochemistry. 

Correlation analyses showed a positive association between leptin and three classical parameters related to glycemic control, serum glucose, serum insulin and HOMA-IR index, and negative correlations between each of these parameters and adiponectin or FGF21. The correlations between leptin or adiponectin and glycemic control-related parameters have been widely described [15,16,17,18,19], and the present results are in accordance with the published works. Few clinical studies examining the effects of FGF21 on glycemic control and obesity are available. In the present work, increased serum FGF21 levels showed a positive correlation with glycemic control-related parameters. Nevertheless, the multiple regression analysis did not show any association between this adipokine and the glycemic control-related parameters. 

In general, FGF21 influences glucose homeostasis by stimulating glucose uptake by adipocytes and inhibiting glucose production in the liver or improving insulin sensitivity [20]. In accordance with our results, other authors observed higher serum FGF21 levels in subjects with obesity compared to those detected in lean individuals [21,22]. However, a recent meta-analysis revealed that the use of FGF21 analogs exhibited no effect on fasting blood glucose, glycated hemoglobin, and HOMA index, although a decreased fasting insulinemia was detected. As stated by the authors, the quality of the evidence ranged from moderate to very low and more clinical trials are needed to increase the quality of this evidence [23].

Regarding serum lipids, the correlation study showed significant correlations with adiponectin, leptin and FGF21. The regression analysis confirmed the positive association between FGF21 and triglycerides and a negative correlation between FGF21 and HDL-cholesterol, which is in line with that observed by other authors [24,25,26]. It has been shown that this cytokine increases the expression of PGC1-α in the liver, which induces FFA oxidation through mitochondrial enhancement, thus preventing their conversion into triglycerides and reducing serum triglyceride concentration. Moreover, FGF21 lowers serum triglyceride levels through the suppression of white adipose tissue lipolysis, lipoprotein lipase activity increase and thus lipoprotein clearance enhancement [27]. Taking these facts into account, a resistance to FGF21 can be proposed [28,29]. Regarding, HDL-cholesterol, it has been demonstrated that FGF21 inhibits the expression of SREBP-2 [30], and that this transcription factor activates the transcription of the hepatic enzyme ABCA1, which catalyzes the assembly of cholesterol, phospholipids and apoproteins for HDL-c secretion from liver [31]. Consequently, FGF21 reduces HDL-cholesterol production. Moreover, a negative association between adiponectin and serum triglycerides, and a positive association between adiponectin and HDL-cholesterol were also confirmed by the regression analysis. These results are in accordance with those reported by other authors [32,33]. With regard to the negative association between adiponectin and serum triglycerides, it has been proposed that adiponectin increases triglycerides clearance by increasing lipoprotein lipase expression in white adipose tissue [34]. The positive association between adiponectin and HDL-cholesterol can be explained because compelling evidence suggests that adiponectin plays a significant role in promoting cellular cholesterol efflux and HDL biogenesis. Indeed, it has been hypothesized that ABCA1 activity and adiponectin receptors, AdipoR1 and AdipoR2, play a critical role in adiponectin-mediated cholesterol efflux [35].

In addition, adiponectin was negatively correlated with uric acid. Other studies in the literature have also shown this correlation in cohorts with characteristics different from those of the present study, in terms of ethnic background, age or gender [36,37,38]. However, the multiple regression analysis did not confirm the association. 

Although it is known that transaminases are not as good markers of liver damage as imagined, ALT is the most commonly used parameter to reflect hepatic impairment, including non-alcoholic fatty liver disease (NAFLD). Other authors reported a positive correlation between ALT and FGF21 [39,40]. Moreover, several studies have shown that plasma FGF21 concentration was highly correlated with hepatic fat content in cases of mild and moderate liver steatosis, either in adult subjects [41,42,43,44] or children [45,46,47,48]. Unfortunately, once again the multiple regression analysis did not confirm the association. In contrast, regression analysis showed a positive association between leptin and the ratio ALT/AST. Taking into account that leptin increases in overweight subjects and subjects with obesity, this association could suggest the development of fatty liver in the subjects with obesity in the present study. Nevertheless, this hypothesis could not be confirmed because, unfortunately, potential liver steatosis was not analyzed in these subjects. 

To summarize, the main result concerning the relationship between serum biochemical parameters and serum cytokines are the significant associations found between FGF21 and adiponectin with serum lipids (triglycerides and HDL-cholesterol).

With regard to the fatty acid profile in RBC membranes, in subjects with obesity this was characterized by an increase in total ω-6 fatty acids, as a result of a higher level of DGLA, and a decrease in total ω-3 fatty acids, mainly due to a lower level of DHA. Consequently, the ratio ω-6/ω-3 was significantly higher. The increase in ω-6 fatty acids and the decrease in ω-3 fatty acids observed in subjects with obesity, when compared with normal-weight subjects, indicated that the former showed an inflammatory status, as was also suggested by a reduced adiponectin level.

Concerning DGLA, several authors have proposed that the increased level of this fatty acid observed in obesity may represent a mechanism conferring protective effects against the associated inflammation [49,50]. This is due to its conversion into the series-1 prostanoids via the cyclooxygenase pathway and suppression of inflammatory leukotriene (LT) formation, and the ability to compete with AA in the synthesis of pro-inflammatory AA mediators [51,52]. However, other studies have reported a positive association with inflammatory and endothelial activation markers, with a more significant association found in obese adults [53]. In fact, DGLA can be converted to 15-HETrE via the 15-lipoxygenase pathway [54]. Regression analysis revealed a negative association between DGLA and adiponectin, suggesting that the increase in DGLA in RBC membranes acts as an inflammatory marker [51].

On the other hand, DHA, an ω-3 polyunsaturated fatty acid with cardioprotective effects was negatively associated with leptin. In fact, low levels of ω-3 fatty acids and high levels of leptin have been reported in subjects with obesity, which are related to a proinflammatory state [55]. A recent systematic review revealed that an increase in DHA intake can negatively modulate the expression of leptin [56]. Taking into account that RBC fatty profiles reflect in part fatty acid intake, this result might be in line with the association found in the present study. 

The association between serum triglycerides and the content of DGLA in RBC has been scarcely studied. The present results do not agree with those reported by Deon et al., who did not find any significant association, but it should be noted that their study was addressed in children and adolescents with primary hyperlipidemia [57]. 

In addition, adiponectin was positively correlated with ∆6D + ELO and the association was maintained after the adjustment in the multiple regression analysis. There are few studies showing this association in the literature; they have been addressed, not in RBC membrane phospholipids, but in plasma phospholipids [58,59]. Nevertheless, taking into account that plasma phospholipids do not mirror RBC membrane phospholipids [60,61], no comparisons with the present results can be made.

In conclusion, important alterations in serum adipokine profile and RBC fatty acid profiles are found in subjects with obesity. Regression analysis reveals interesting associations between adipokines and other biochemical serum parameters, between RBC fatty acid profiles and general biochemical serum parameters, and between serum adipokines and RBC fatty acid profile, some of them being described for the first time. In view of these associations, further studies are needed to better explain them, and to check if they are also found in other cohorts. 

The present study shows some limitations, the relatively small number of subjects in the sample being one of them. This fact may have affected the strength of the correlations found in this cohort. Another limitation is that, due to the cross-sectional nature of the study, it is not possible to determine a cause-and-effect relationship between changes in adipokine and RBC fatty acid profiles and the alterations found in the serum biochemical parameters in the obese subjects. Moreover, diet is considered to be the major modifying factor of fatty acid composition of tissues. Unfortunately, dietary intake was not registered in the present study.

## Figures and Tables

**Table 1:** Anthropometric parameters in normal-weight subjects and subjects with obesity.

	Normal-Weight Subjects (n = 37)	Subjects with Obesity (n = 38)
Weight (kg)	66.9 ± 1.4	111.6 ± 5.0 ***
Height (cm)	170.4 ± 1.7	166.9 ± 1.60
BMI (kg/m^2^)	22.9 ± 0.2	40.8 ± 1.1 ***
Waist circumference (cm)	78.7 ± 1.6	120.0 ± 2.6 ***

Data are expressed as mean ± SEM. *** *p* < 0.001. BMI: body mass index.

**Table 2:** Biochemical parameters in normal-weight and obese subjects.

	Normal-Weight Subjects (n = 37)	Subjects with Obesity (n = 38)
Glucose (mg/dL)	86.9 ± 1.50	103.0 ± 3.2 **
Insulin (mU/L)	10.0 ± 1.1	20.5 ± 1.9 **
HOMA-IR	2.2 ± 0.2	5.6 ± 0.5 **
Total cholesterol (mg/dL)	184.8 ± 6.6	193.4 ± 5.3
HDL-c (mg/dL)	61.3 ± 2.6	50.5 ± 2.7 **
LDL-c (mg/dL)	107.1 ± 5.3	121.9 ± 4.9 *
Triglycerides (mg/dL)	80.72 ± 4.9	155.5 ± 11.2 **
AST/GOT (U/L)	19.3 ± 0.8	21.7 ± 1.3
ALT/GPT (U/L)	19.5 ± 1.7	26.3 ± 1.9 *
AST/ALT	1.2 ± 0.1	0.9 ± 0.04 **

Data are expressed as mean ± SEM. * *p* < 0.05; ** *p* < 0.01. ALT/GPT: Alanine transaminase; AST/GOT: Aspartate aminotransferase; HDL-c: high-density lipoprotein cholesterol; HOMA-IR: homeostasis model of assessment of insulin resistance; LDL-c: low-density lipoprotein cholesterol.

**Table 3:** Red blood cell (RBC) membrane fatty acid profile in normal-weight subjects and subjects with obesity.

Fatty Acid (%)	Normal-Weight Subjects (n = 37)	Subjects with Obesity (n = 38)
Palmitic acid (C16:0)	22.13 ± 0.27	22.66 ± 0.19
Stearic acid (C18:0)	17.12 ± 0.23	17.11 ± 0.19
Total SFAs	39.25 ± 0.29	39.77 ± 0.22
Sapienic acid (C16:1, 6c)	0.38 ± 0.03	0.32 ± 0.04
Palmitoleic acid (C16:1, 9c)	0.48 ± 0.03	0.45 ± 0.03
Oleic acid (C18:1, 9c)	17.04 ± 0.19	16.75 ± 0.22
*cis*-Vaccenic acid (C18:1, 11c)	1.29 ± 0.05	1.24 ± 0.04
Total MUFAs	19.19 ± 0.21	18.76 ± 0.23
Linoleic acid (C18:2)	12.82 ± 0.20	12.43 ± 0.29
Alpha-linolenic acid (C18:3)	0.22 ± 0.02	0.20 ± 0.02
Dihomo-γ-linolenic acid (C20:3)	1.84 ± 0.09	2.19 ± 0.07 **
Arachidonic acid (C20:4)	18.03 ± 0.33	18.89 ± 0.31
Eicosapentaenoic acid (C20:5)	0.73 ± 0.08	0.64 ± 0.06
Docosapentaenoic acid (C22:5)	1.97 ± 0.07	1.82 ± 0.05
Docosahexaenoic acid (C22:6)	5.75 ± 0.20	5.12 ± 0.22 *
Total ω-6	32.69 ± 0.46	33.50 ± 0.40
Total ω-3	8.67 ± 0.29	7.80 ± 0.30 *
Total PUFA	41.36 ± 0.42	41.30 ± 0.31
*Trans* C18:1	0.09 ± 0.01	0.10 ± 0.01
*Trans* C20:4	0.11 ± 0.02	0.08 ± 0.01
Total *Trans*	0.20 ± 0.02	0.18 ± 0.02
SFA/MUFA	2.05 ± 0.02	2.13 ± 0.03 *
ω-6/ω-3	3.95 ± 0.16	4.56 ± 0.20 *
∆6D + ELO 20:3/18:2	0.15 ± 0.01	0.18 ± 0.01 *
∆5D 20:4/20:3	10.47 ± 0.45	8.98 ± 0.35 *
∆9D 16:1/16:0	58.88 ± 6.12	64.49 ± 6.12
∆9D 18:1/18:0	1.01 ± 0.02	1.03 ± 0.02

Data are presented as mean ± standard error of the mean. * *p* < 0.05; ** *p* <0.01. D: desaturase; ELO: elongase; MUFA: monounsaturated fatty acid; PUFA: polyunsaturated fatty acid; SFA: saturated fatty acid.

**Table 4:** Correlations between serum cytokines and biochemical parameters.

		Adiponectin	NOV/CCN3	Leptin	FGF21
Glucose	ρ	−0.338 **	0.146	0.453 **	0.356 **
	*p*	**0.004**	0.244	**0.000**	**0.002**
Insulin	ρ	−0.392 **	0.061	0.495 **	0.451 **
	*p*	**0.001**	0.615	**0.000**	**0.000**
HOMA-IR	ρ	−0.441 **	0.079	0.504 **	0.503 **
	*p*	**0.000**	0.513	**0.000**	**0.000**
Triglycerides	ρ	−0.434 **	0.223	0.395 **	0.546 **
	*p*	**0.000**	0.066	**0.001**	**0.000**
Cholesterol	ρ	0.144	0.119	0.176	0.123
	*p*	0.221	0.325	0.133	0.295
HDL-c	ρ	0.441 **	−0.062	−0.179	−0.389 **
	*p*	**0.000**	0.624	0.142	**0.001**
LDL-c	ρ	0.027	0.213	0.198	0.229
	*p*	0.831	0.094	0.109	0.063
Uric acid	ρ	−0.360 **	0.174	0.196	0.313 **
	*p*	**0.002**	0.150	0.095	**0.007**
ALT/AST	ρ	−0.353 **	0.012	0.027	0.265 *
	*p*	**0.003**	0.923	0.821	**0.026**

ρ: Spearman’s correlation coefficient; *p*: *p*-value. * *p* < 0.05; ** *p* < 0.01. Significant correlations are in bold. ALT: Alanine transaminase; AST: Aspartate aminotransferase; HDL-c: high-density lipoprotein cholesterol; HOMA-IR: homeostasis model of assessment of insulin resistance; LDL-c: low-density lipoprotein cholesterol.

**Table 5:** Correlations between fatty acids in RBC membranes and cytokines.

		Adiponectin	Leptin	FGF21
∆6D + ELO	ρ	0.412 **	−0.344 *	−0.322 *
	*p*	**0.004**	**0.024**	**0.042**
∆5D	ρ	0.374 *	−0.197	−0.263
	*p*	**0.011**	0.301	0.121
DGLA	ρ	−0.378 *	0.068	0.337 *
	*p*	**0.011**	0.297	**0.029**
DHA	ρ	0.177	−0.375 *	−0.131
	*p*	0.364	**0.011**	0.531
total ω-3 fatty acids	ρ	0.122	−0.393 **	−0.168
	*p*	0.573	**0.006**	0.398
ω-6/ω-3 ratio	ρ	−0.124	0.345 *	0.157
	*p*	0.562	**0.024**	0.435

Ρ: Spearman’s correlation coefficient; *p*: *p*-value. * *p* < 0.05; ** *p* < 0.01. Significant correlations are in bold.; D: desaturase; DGLA: dihomo-γ-linolenic acid; DHA: docosahexaenoic acid; ELO: elongase.

**Table 6:** Stepwise multiple regression analysis.

Dependent Variable	Independent Variables	β Coefficient	*p*	Adjusted R^2^
HOMA-IR	sex	−0.251	0.040	0.362
	adiponectin	−0.273	0.017	
	leptin	0.521	0.000	
Triglycerides	FGF21	0.476	0.000	0.329
	adiponectin	−0.277	0.007	
HDL-c	FGF21	−0.272	0.017	0.207
	adiponectin	0.360	0.002	
Uric acid	age	0.267	0.012	0.236
	sex	−0.420	0.000	
ALT	sex	−0.553	0.000	0.296
Adiponectin	HOMA-IR	−0.320	0.005	0.352
	sex	0.307	0.004	
	D6D + ELO	0.315	0.005	
Leptin	HOMA-IR	0.175	0.045	0.876
	age	0.175	0.009	
	sex	0.520	0.000	
	ALT/AST	0.128	0.021	
	DHA	−0.175	0.000	
FGF21	triglycerides	0.329	0.003	0.339
	age	0.330	0.003	
	HDL-cholesterol	−0.232	0.029	
DGLA	triglycerides	0.312	0.015	0.187
	adiponectin	−0.253	0.047	
Δ6D + ELO	adiponectin	0.321	0.008	0.283
	leptin	−0.426	0.001	
Δ5D	triglycerides	−0.410	0.000	0.156

HDL-c: high-density lipoprotein cholesterol; HOMA-IR: homeostasis model of assessment of insulin resistance; ALT: Alanine transaminase; AST: Aspartate aminotransferase; DHA: Docosahexaenoic acid; D: desaturase; DGLA: Dihomo-γ-linolenic acid; ELO: elongase; FGF21: fibroblast growth factor 21. Sex variables considered as 0 for males and 1 for females.

## Data Availability

The data that support the findings of this study are available from the corresponding author, upon reasonable request.
